# A Multifunctional Joint Angle Sensor with Measurement Adaptability

**DOI:** 10.3390/s131115274

**Published:** 2013-11-08

**Authors:** Wei Quan, Hua Wang, Datong Liu

**Affiliations:** 1 School of Transportation Science and Engineering, Harbin Institute and Technology, Harbin 150090, China; E-Mail: hitwh@hit.edu.cn; 2 Department of Automatic Test and Control, Harbin Institute and Technology, Harbin 150080, China; E-Mail: liudatong@hit.edu.cn

**Keywords:** joint angle, wearable device, multifunctional sensor, gesture measurement

## Abstract

The paper presents a multifunctional joint sensor with measurement adaptability for biological engineering applications, such as gait analysis, gesture recognition, *etc.* The adaptability is embodied in both static and dynamic environment measurements, both of body pose and in motion capture. Its multifunctional capabilities lay in its ability of simultaneous measurement of multiple degrees of freedom (MDOF) with a single sensor to reduce system complexity. The basic working mode enables 2DOF spatial angle measurement over big ranges and stands out for its applications on different joints of different individuals without recalibration. The optional advanced working mode enables an additional DOF measurement for various applications. By employing corrugated tube as the main body, the sensor is also characterized as flexible and wearable with less restraints. MDOF variations are converted to linear displacements of the sensing elements. The simple reconstruction algorithm and small outputs volume are capable of providing real-time angles and long-term monitoring. The performance assessment of the built prototype is promising enough to indicate the feasibility of the sensor.

## 1. Introduction

Measurement of human pose and motion generates interest among researchers because of their wide applications in fields such as gait analysis [1], gesture recognition [2], and motion capture [3] for the purposes such as clinical research, rehabilitation, vehicle control, professional sports training, *etc.* In both pose and motion analysis, the human anatomy is frequently represented as a sequence of rigid bodies or links, connected by joints for analytical convenience. Joints are mainly classified structurally and functionally, which can also be classified according to the degrees of freedom (DOF) allowed, and distinguished between joints with one, two or three degrees of freedom. In most literature on topics such as gait analysis, gesture recognition, *etc.*, various approaches are presented for determining angular parameters of joints, such as the knee, ankle, shoulder, elbow, *etc.* as the most significant aims. This study focuses on developing a joint angle sensor capable of measuring multiple degrees of freedom (MDOF) with measurement adaptability.

Inertial measurement units (IMUs), compact wearable devices that contain a triaxial accelerometer and a triaxial gyroscope, are some of the most popular devices used to sense movement and orientation of the moving body that can help calculate joint angles because of their capability of reconstructing the trajectory of sensed anatomical points. Kinematic values such as shank and thigh inclination angles, knee joint angles or elbow joint angles can be derived by integration of angular acceleration or angular velocity [4–8]. But IMUs face a problem in that the data obtained from integration can be distorted by offsets or drifts, which result the fact that inaccuracies inherent in the measurements quickly accumulate and rapidly degrade the accuracy. Especially if a subject is contained in a dynamic environment, such as a moving vehicle or aeroplane, the magnitude and orientation of the measured acceleration vectors may vary significantly with time. Some literatures combine a triaxial accelerometer, a triaxial gyroscope and a triaxial magnetometer assembled to produce a sensor module referred to as a Magnetic, Angular Rate and Gravity sensor (MARG). Two tri-axial accelerometers, two tri-axial rate gyroscopes, two tri-axial magnetometers are employed to obtain the 3D ankle joint angle in [9]. Orientation and position were obtained by single and double integration of gyroscope and accelerometer data, respectively. These were then updated with magnetometer data to improve the accuracy [10]. Nowadays, various literatures are focused on alleviating of the cumulative drift, increasing the measurement accuracy, long-term application and MDOF achievement [11–15].

Vision-based systems are another popular method for tracking motion and recognizing the pose of humans. Single or multiple cameras acquire video streams that are processed and gestures are mapped into temporal signatures of changes in video frames [16–18]. Passive/Active marker systems place a number of dots strategically on the human body to perceive the position, movement and other information about bodies [19]. Inverse kinematics is commonly employed to determine the joint parameters by the markers' position. The major advantage of optical systems is that they do not need wires and tethers, however, vision-based systems face problems such as contending with occlusions which may results in losses of some motion information, and the requirements of the monitoring conditions such as brightness, background, *etc.* Therefore, they are not suitable to capture movement information over a larger distance and outside a laboratory environment.

Additionally, methods for joint angle measurement based on other principles were also proposed. The main problem of the bio-measurement method [20] is that the absolute impedance values and their changes differ between individuals. Johnson [21] developed an implantable transducer for 2DOF joint angle sensing, whose invasive character may be not suitable for some persons.

This study aims to develop a joint angle sensor with measurement adaptability. The adaptability is embodied in suiting both static and dynamic environment measurements, both pose and motion capture, and the capability of simultaneously measuring MDOF with a single sensor to reduce system complexity. It is preferable for the sensor to be flexible for measurement of multi-axial joints, such as shoulders, ankles, *etc.* The paper presents a novel sensor without the issues mentioned above and mainly contributes to the following aspects:

(1) Capability of measuring 3DOF simultaneously with two working modes. The basic mode works for achieving orientation and obliquity of a three-dimensional (3D) joint. The optional advanced mode is able to measure an additional DOF.
(2) The basic mode enables the applications on different joints of different individuals without recalibration.
(3) Large ranges of measurement for freely arbitrary movement. The orientation is over the range 360°. The ranges of obliquity and additional angle can be designed by requirement.
(4) Pose and motion capture in both static and dynamic environments.
(5) Utilization of the minimum number of outputs for reconstructing three angular parameters. This will greatly reduce the volume of testing data compared to other approaches. The simple reconstruction algorithm and small data volume are capable of providing real-time angles and long-term monitoring.
(6) Other characteristics such as flexibility and insensitivity to environment disturbances.

## 2. Working Principle

**Figure 1.**
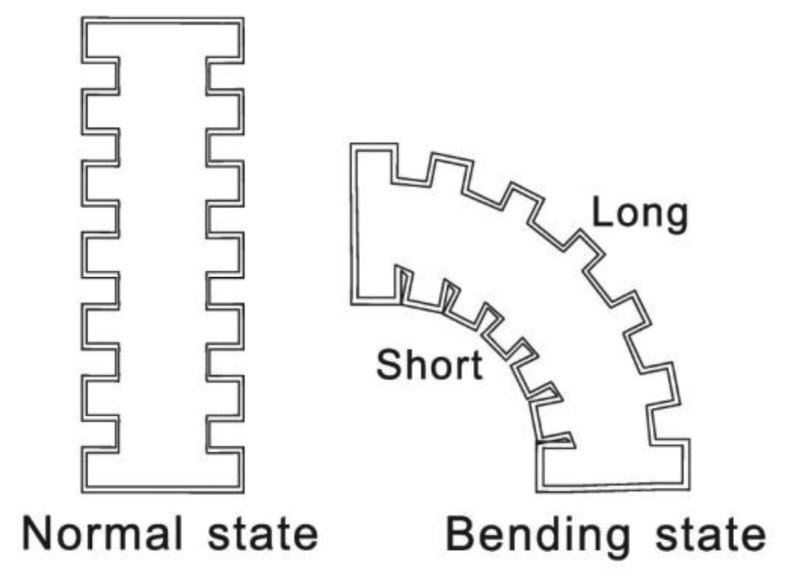
Working principle and corrugated tube character.

Skin is an organ of the integumentary system that protects the underlying muscles and organs. It is flexible and has many wrinkles, especially on the joints. Movement of extremities cause the corresponding extension/accumulation of wrinkles. If we draw several black lines on the elbow, their length will differ with different joint motions, thus the angular parameters are converted into length changes, but it is difficult to measure the actual length of skin and it differs between individuals. A corrugated tube shaped into alternating parallel grooves and ridges is selected as the sensor body to imitate the action of skin, as illustrated in Figure 1.

Two important characteristics of the tube are keeping the constant radius of the ridges and resisting twisting. The tube ends are mounted on the two segments of the tested object, whose relative position indicates the 3D joint spatial status.

### 2.1. Working Coordinate System

The multi-axial joints of the human body are mainly the wrist, shoulder, hip and ankle. Two angular parameters can describe their spatial relative position. Among them, the shoulder and hip joints can be similar sketched as ball and socket joints with three DOF. Generally, the Euler angles are three angles introduced by Euler to describe the orientation of a rigid body. They are the azimuth *ϕ* of the top about the vertical; the tilt angle *θ* between the symmetry axis of the top and the vertical; and the rotation angle *ψ* of the top about its own axis. In rigid body dynamics, precession is the change in *ϕ*, nutation is the change in *θ* and rotation is the change in *ψ*.

**Figure 2.**
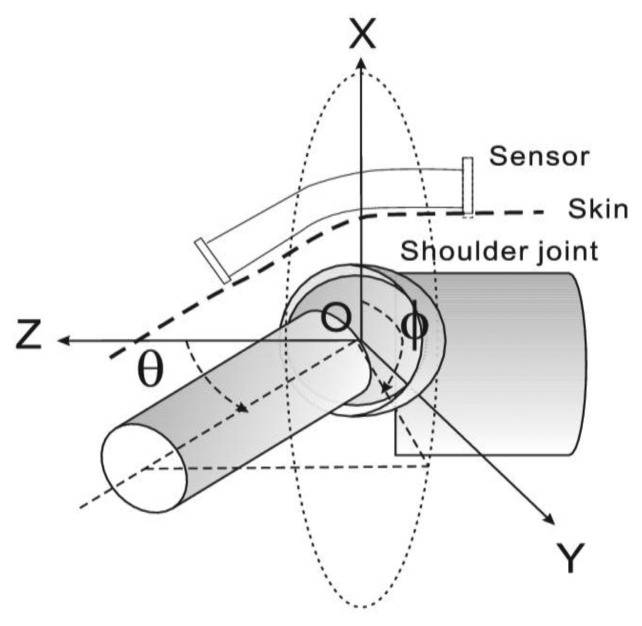
Skeleton of shoulder joint and definitions of 2DOF.

Figure 2 illustrates the skeleton structure of a shoulder joint and defines the reference frame and two angular parameters. The shoulder joint is taken as an example to introduce the coordinate system for the reason that it is the most mobile joint in the body (the reasoning can easily be extended to other cases). For the shoulder joint, a rotation angle exists, which is led by the rotator cuff muscles attached on the proximal humerus and is a gradual change from the shoulder joint to the elbow. The mounting of the tube end on the deltoid muscle area efficiently reduces the effect caused by rotation because of the slight transverse deformation of skin on the deltoid muscle. The estimation of rotation is introduced as the advanced mode in the later section. Tube ends are mounted on the two segments and form the corresponding frames separately. *ϕ* is the precession angle from the positive x-axis which indicates the bending orientation of the arm. *θ* is the nutation angle from the positive z-axis which is the obliquity of the arm. The estimation of *ϕ* and *θ* is the basic mode in this study.

### 2.2. Geometrical Analysis

**Figure 3.**
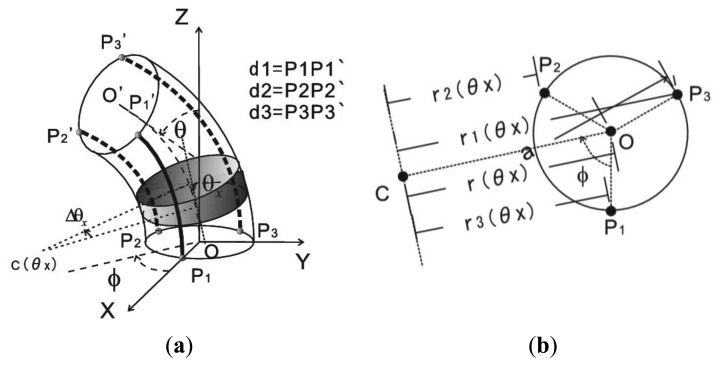
Geometric analysis (**a**) Working coordinate system of sensor; (**b**) Calculation of bending radius of three lateral lines.

According to Figure 2, the working coordinate system of the tube for geometrical analysis is illustrated in Figure 3. Three generatrices (*P*_1_*P*_1_*'*, *P*_2_*P*_2_*'* and *P*_3_*P*_3_*'*) with 120° angular separation are selected to calculate *ϕ* and *θ*. The initial position of sensor in two segments keeps in a straight line with obliquity *θ* that equals zero. These lines change gradually with tilting of one segment. *C*(*θ*_x_) is the bending central point of the tube central axis with the obliquity as *θ*_x_. *d*_1_, *d*_2_ and *d*_3_ represent the length of *P*_1_*P*_1_*'*, *P*_2_*P*_2_*'* and *P*_3_*P*_3_*'*. They are calculated by the definite integral of arc lengths with interval of 0° to *θ*:

(1) $$d_{1,2,3}=\int_{0}^{\theta }r_{1,2,3}\;(\theta_{x})d\theta_{x}$$

where *r*_1,2,3_(*θ*_x_) are the bending radius of three lines that are parallel to *OC*, as shown in Figure 3b.

They are the functions of orientation *ϕ*:

(2) $$\{\begin{array}{l} r_{1}(\theta_{x})=r(\theta_{x})-a\;\;\cdot \;\;\cos\phi \\ r_{2}(\theta_{x})=r(\theta_{x})-a\;\;\cdot \;\;\cos(\phi -\frac{2\pi }{3})\\ r_{3}(\theta_{x})=r(\theta_{x})-a\;\;\cdot \;\;\cos(\phi +\frac{2\pi }{3}) \end{array}$$

where *r*(*θ*_x_) is the bending radius of the tube central axis that changes gradually with obliquity, and *a* is the radius of the tube.

Substituting Equation (2) into Equation (1), *d*_1_, *d*_2_ and *d*_3_ are expressed as:

(3) $$\left\{ \begin{array}{l} d_{1}=\int_{0}^{\theta }r(\theta_{x})d\theta_{x}-a\;\;\cdot \;\;\cos\phi \;\cdot \;\theta \\ d_{2}=\int_{0}^{\theta }r(\theta_{x})d\theta_{x}-a\;\cdot \;\cos(\phi -\frac{2\pi }{3})\;\cdot \;\theta \\ d_{3}=\int_{0}^{\theta }r(\theta_{x})d\theta_{x}-a\;\cdot \;\cos(\phi +\frac{2\pi }{3})\;\cdot \;\theta \end{array}\right.$$

The subtraction of each two equations will eliminate the integral part. *ϕ* and *θ* can be easily calculated by the known *d*_1_, *d*_2_ and *d*_3_ and radius *a*:

(4) $$\left\{ \begin{array}{l} d_{1}-d_{2}=\sqrt{3}\;\cdot \;a\;\cdot \;\sin(\phi -\frac{\pi }{3})\;\cdot \;\theta \\ d_{1}-d_{3}=-\sqrt{3}\;\cdot \;a\;\cdot \;\sin(\phi +\frac{\pi }{3})\;\cdot \;\theta \end{array}\right.$$

## 3. Configuration of the Sensor Prototype and Two Working Modes

### 3.1. Configuration of the Prototype

**Figure 4.**
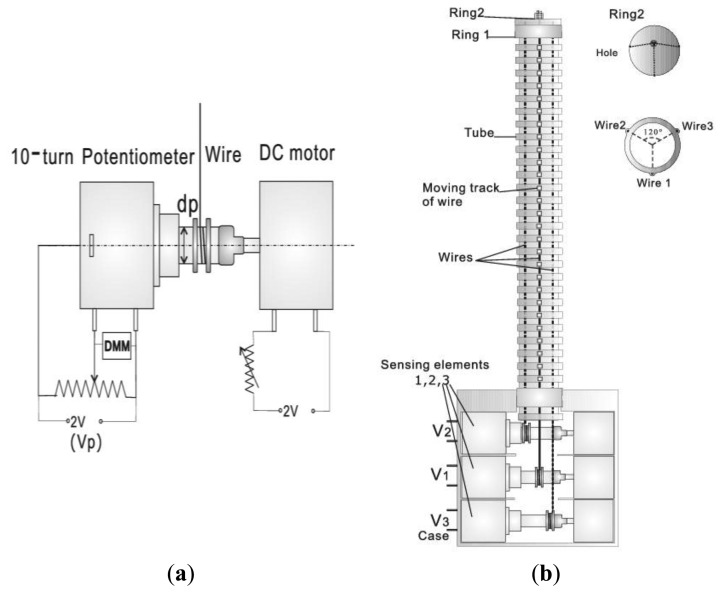
Configuration of the prototype. (**a**) Sensing element; (**b**) Structure of the device.

Based on the introduced principle, a sensor capable of measuring the surface lengths of corrugated tube is proposed. A prototype is built to test the feasibility of the method. Its configuration is shown in Figure 4. A flexible and corrugated tube with external radius in 9 mm and length in 200 mm is selected as the main body. Three tracks composed of several small rings with radius in 1 mm are built on the ridge surface of the tube with 120° angular separation of each other, which enables the smooth movement of iron wires on the tube surface in a fixed lateral orbit. The tube is capable of extending/compressing at different angular position, whose pose is reflected by the displacement of three wires.

The sensing element prototype is built to test the wire displacement, which is mainly composed of a stainless wire, a 10-turn rotary potentiometer and a DC motor, as shown in Figure 4a. The potentiometer has a 5 kΩ range. 2 V DC voltage is applied as the fixed input voltage across the two ends of the pots. One end of the wire is fixed and wound about four turns on the shaft of potentiometer as the initial position with 1 V output. The DC motor connected with the potentiometer produces a suitable strain to draw back the lax wire. Therefore, the wire displacement can be measured by the potentiometer with a linear output. Three sensing elements are employed to provide three DC voltages outputs.

Wires of three sensing elements run through the tracks built on the flexible tube, respectively. Their free ends are fixed on the tube by ring 1 and then clustered into one bundle by the small hole on ring 2. The design of ring 1 and ring 2 is used to induce the optional advanced working mode. The end of the flexible tube and three testing segments are mounted in a case as shown in Figure 4b.

Movement of the wire leads to the rolling of the potentiometer to represent a voltage divider. Three DC voltages *V*_1_, *V*_2_ and *V*_3_ are measured as outputs by a digital multimeter (DMM). Three motors used to roll back the potentiometers are driven by a DC voltage source of 2 V which is able to control the strain on the wires. In order to achieve the characteristics and prove the feasibility of the proposed sensor structure, an experimental platform is built, which is a mechanical frame imitating a joint with two freedoms of motion, containing orientation scale and obliquity scale. Two ends of the prototype are fixed on the frame upper side and under side. By accurately adjusting the relative position of two ends of the frames with scales, the prototype can be set to the required positions with orientation *ϕ* over the range of 360° and obliquity *θ* over the range of 90°.

The purpose of fabricating the prototype is only for proving the feasibility of the proposed method. The basic configuration and working principle of proposed sensor are more meaningful than the detailed structure of prototype. The size of the actual device can be designed freely depending on the tested object. The tube radius is the most important and the only required parameter for the subsequent estimation process.

### 3.2. Basic Mode: Measurement of Obliquity and Orientation

The device outputs can be expressed as *V*_1,2,3_= *V*_0_+ Δ*V*_1,2,3_, where *V*_0_ is the output at initial position and Δ*V*_1,2,3_ are the variations of *V*_1,2,3_. The relations of three voltages and displacement of wires is expressed as below:

(5) $$\begin{array}{cc} \Delta V_{1,2,3}=k_{v}\Delta d_{1,2,3} & \left(k_{v}=\frac{V_{p}}{10\pi d_{p}}(mv/mm)\right) \end{array}$$

where *k*_v_ is the voltage change with 1 mm displacement of wire which is a constant only decided by the potentiometer and its shaft size. We suppose *d*_1,2,3_ = *d*_0_ + Δ*d*_1,2,3_, where *d*_0_ is the length of wires at initial position, and Δ*d*_1,2,3_ are the displacements separately. From Equations (4) and (5), Equation (6) is achieved with *ϕ* and *θ* as two unknown variables, which appears a very simple format. *k*_v_ and *a* are the only required parameters to calculate *ϕ* and *θ*. It enables the adaptable applications on different joints of different individuals without recalibration and a simple reconstruction algorithm:

(6) $$\left\{ \begin{array}{l} \Delta V_{1}-\Delta V_{2}=\sqrt{3}\;\cdot \;k_{v}\;\cdot \;a\;\cdot \;\sin(\phi -\frac{\pi }{3})\;\cdot \;\theta \\ \Delta V_{1}-\Delta V_{3}=\sqrt{3}\;\cdot \;k_{v}\;\cdot \;a\;\cdot \;\sin(\phi +\frac{\pi }{3})\;\cdot \;\theta \end{array}\right.$$

### 3.3. Advanced Mode: Measurement of an Addition Angle

Besides the measurement of *ϕ* and *θ* as the basic mode, the proposed sensor is capable of measuring an additional angle as the advanced mode. The advanced mode is optional and needs a precalibration to build output equations for different individuals. The reconstruction algorithm of the additional DOF is relative complex, but the advanced mode provides the multifunctional capability, which can reduce the physical system complexity and data volume.

#### 3.3.1. Working Principle of the Advanced Mode

In the basic mode, *ϕ* and *θ* are calculated by the difference of three outputs. Substituting Equation (5) into Equation (3), Equation (7) is obtained with two unknown variables:

(7) $$\begin{array}{l} \left\{ \begin{array}{l} \Delta V_{1}=k_{v}\;\cdot \;(\int_{0}^{\theta }r(\theta_{x})d\theta_{x}-d_{0})-k_{v}\;\cdot \;a\;\cdot \;\cos\phi \;\cdot \;\theta \\ \Delta V_{2}=k_{v}\;\cdot \;(\int_{0}^{\theta }r(\theta_{x})d\theta_{x}-d_{0})-k_{v}\;\cdot \;a\;\cdot \;\cos(\phi -\frac{2\pi }{3})\;\cdot \;\theta \\ \Delta V_{3}=k_{v}\;\cdot \;(\int_{0}^{\theta }r(\theta_{x})d\theta_{x}-d_{0})-k_{v}\;\cdot \;a\;\cdot \;\cos(\phi +\frac{2\pi }{3})\;\cdot \;\theta \end{array}\right.\\ g(\phi ,\theta )=k_{v}\;\cdot \;\left(\int_{0}^{\theta }r(\theta_{x})d\theta_{x}-d_{0}\right) \end{array}$$

Generally, three uncorrelated equations have the capability to calculate three unknown variables. The attachment of sensor on different individuals produces different expressions *g*(*ϕ*, *θ*). The same Γ(*ψ*) is added to each equation of Equation (7). Three equations with three variables are then expressed as in Equation (8):

(8) $$\left\{ \begin{array}{l} \Delta V_{1}=g(\phi ,\theta )-k_{v}\;\cdot \;a\;\cdot \;\cos\phi \;\cdot \;\theta +\Gamma (\psi )\\ \Delta V_{2}=g(\phi ,\theta )-k_{v}\;\cdot \;a\;\cdot \;\cos(\phi -\frac{2\pi }{3})\;\cdot \;\theta +\Gamma (\psi )\\ \Delta V_{3}=g(\phi ,\theta )-k_{v}\;\cdot \;a\;\cdot \;\cos(\phi +\frac{2\pi }{3})\;\cdot \;\theta +\Gamma (\psi ) \end{array}\right.$$

The subtraction between each two equations of Equation (8) remains the same expression of Equation (6). The additional variable does not affect the basic mode calculation. With the estimated *ϕ* and *θ*, *ψ* can be calculated by each equation in Equation (8). Because the mounting position and motion habit are different for different joints and individuals, a precalibration is needed to build *g*(*ϕ*, *θ*) equation or a database of values before actual applications on individuals.

#### 3.3.2. Applications of the Advanced Mode

In the basic mode, the ends of wires are fixed on the end of the tube by ring 1. In the advanced mode, ring 1 is loosened and the bundle composed of three wires is able to move through the hole on ring 2. The end of the bundle is fixed on the segment of an additional joint, the pose of which is described by *ψ*. It is easy to find that the bundle movement generates the same displacement of the three wires. The advanced mode greatly enlarges the application range of the sensor.

**Figure 5.**
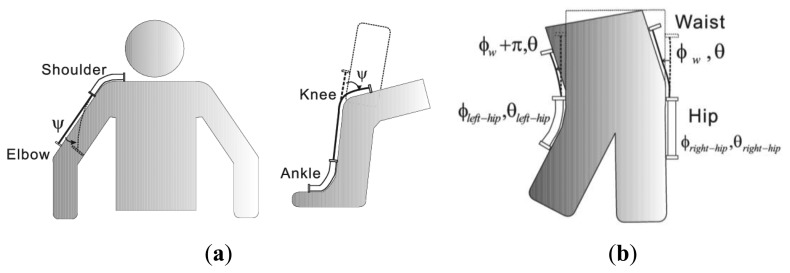
Advanced mode (**a**) Application on an additional unaxial joint angle. Left: Add torsion angle to shoulder joint measurement. Right: Add knee angle to ankle joint; (**b**) Additional measurement of *ϕ*_w_ and *θ*_w_ of waist by employing two hip sensors.

(1) Measurement of an additional uniaxial joint angle
  The application contains two aspects. One is the addition of a traditional uniaxial joint, such as adding a knee angle to an ankle joint measurement or adding the rotation angle of forearm (pivot joint) to the wrist joint. The other one is the additional measurement of another DOF of a joint with 3DOF motion, such as the rotation of a shoulder and hip joint. For the shoulder joint, the rotation angle is led by the rotator cuff muscles attached on the proximal humerus and is a gradual change from the shoulder joint to the elbow. The mounting of tube end on the area of deltoid muscle efficiently reduces the effect caused by rotation. The bundle end is fixed on near the elbow to test the shoulder rotation. Two examples are illustrated in Figure 5a.
(2) Extension for the measurement of an additional 2DOF joint
  The main purpose of the advanced mode lies in reducing the number of sensors mounted on human joints. Besides the measurement of an additional uniaxial joint, the combination of several sensors can realize interesting applications, such as the additional measurements of the waist by employing two advanced mode hip sensors. Figure 5b illustrates this application. The symbols *ϕ*_w_ and *θ*_w_ are used to describe the relative position the ribcage and hips. Two hip sensors are placed on the outboard of the femur and the pelvis, which have a 180° phase different in waist orientation. Γ(*ψ*) is rewritten as Γ(*ϕ*_w_, *θ*_w_) and Γ(*ϕ*_w_ + π, *θ*_w_) for the two sensors. With the calculated value of Γ(*ϕ*_w_, *θ*_w_) and Γ(*ϕ*_w_ + π, *θ*_w_), the *ϕ*_w_ and *θ*_w_ of waist can be reconstructed.

## 4. Experimental Methods, Results and Analysis

### 4.1. Calibration of k_v_

In Equation (6), the only required parameters for calculating *ϕ* and *θ* are *a* and *k*_v_. *k*_v_ is the voltage change with 1 mm displacement of wire and *a* is the tube radius, which are provided by the sensor's physical and electrical structure. Considering the resistance tolerance and linear tolerance of potentiometers, *k*_v_ is calculated by least squares fitting on the experimental results instead of using the product data. Δ*V*_1,2,3_ are measured with the displacement *d* over the range of 40 mm in increments of 2 mm. The fitting slopes are *k*_v1_ = 8.361 mV/mm, *k*_v2_ = 8.429 mV/mm and *k*_v3_ = 8.404 mV/mm, respectively, with a linear tolerance of 0.38%. *k*_v_ is their average value for a simple data process, which is 8.4 mV/mm.

### 4.2. Experimental Results

**Figure 6.**
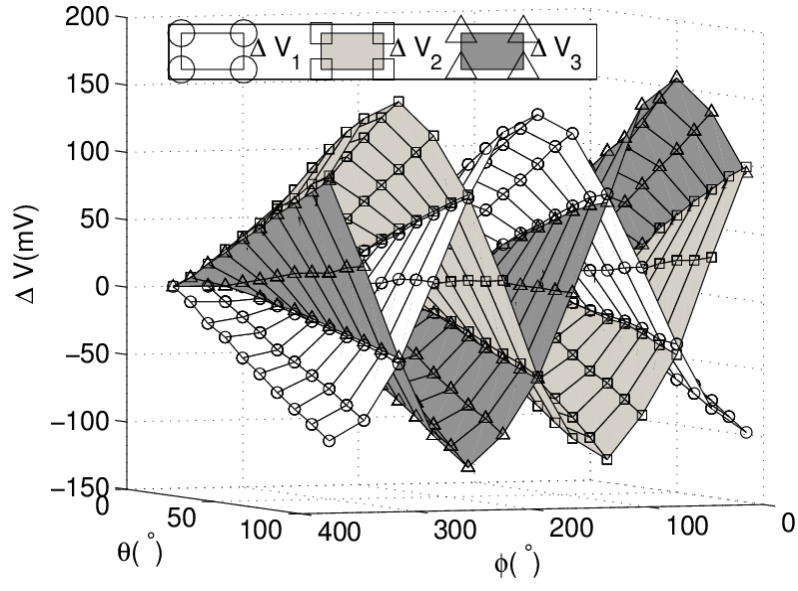
Experimental results on sample points.

Experimental results on sample positions are employed to reconstruct angular parameters and analyze the characteristics of proposed sensor. Sample positions are set with *ϕ* over the range of 360° in increments of 30°, and at each *ϕ* position, measurements are taken for *θ* over the range of 90° in increments of 10° by the experimental platform. *V*_1_, *V*_2_ and *V*_3_ on each sample position are measured in sequence by DMM. Figure 6 shows the surface composed of experimental results of three outputs.

**Figure 7.**
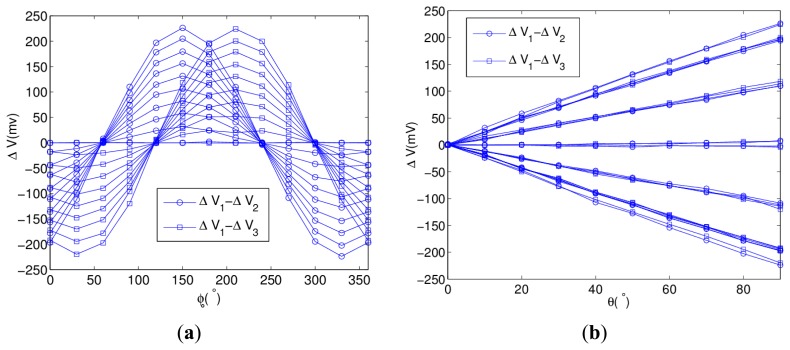
Analysis of sensor outputs (**a**) Characteristic of Δ*V*_1_ − Δ*V*_2_ and Δ*V*_1_ − Δ*V*_3_: Sinusoidal relation with orientation; (**b**) Characteristic of Δ*V*_1_ − Δ*V*_2_ and Δ*V*_1_ − Δ*V*_3_: Linear relation with obliquity.

In Equation (6), the differences of three voltages are used to calculate *ϕ* and *θ*. Figure 7a shows the curves of Δ*V*_1_ − Δ*V*_2_ and Δ*V*_1_ − Δ*V*_3_ with *ϕ* as the variable.

The curves are sinusoidal and their magnitudes depend on the value of obliquity. The phase difference of Δ*V*_1_ − Δ*V*_2_ and Δ*V*_1_ − Δ*V*_3_ is the angular separation of wires. Figure 7b shows the curves of Δ*V*_1_ − Δ*V*_2_ and Δ*V*_1_ − Δ*V*_3_ with *θ* as the variable, which have the linear relation with obliquity at a fixed orientation.

### 4.3. Parameter Reconstruction

**Figure 8.**
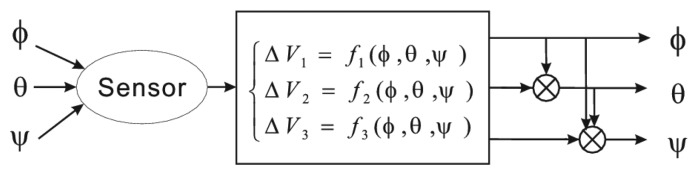
Process of three parameters reconstruction.

This is a ternary function reconstruction problem because each sensing element is cross- sensitive to three angular variables. The process flow of angular parameter reconstruction is shown in Figure 8, which acts in sequence. First *ϕ* is calculated using Equation (6). Then, the estimated *ϕ* is substituted into Equation (6) again to achieve *θ*. If the device works in the advanced mode, *ψ* can be calculated by substituting the estimated *ϕ* and *θ* into Equation (8).

#### 4.3.1. Estimation of Orientation

Orientation can be calculated by dividing each equation of Equation (5), which eliminates *θ* and two constants. We suppose *c* = (Δ*V*_1_ − Δ*V*_2_)/(Δ*V*_1_ − Δ*V*_3_) and *ϕ* can be calculated as below:

(9) $$\begin{array}{l} \phi^{\prime }=\tan^{-1}(\frac{\sqrt{3}(1-c)}{1+c})\\ \left\{ \begin{array}{l} 120{}^{\circ}\begin{array}{cc} & \left(\Delta V_{1}=\Delta V_{3},\Delta V_{2}=\max\Delta V_{1,2,3}\right) \end{array}\\ 300{}^{\circ}\begin{array}{cc} & \left(\Delta V_{1}=\Delta V_{3},\Delta V_{2}\Delta \min\Delta V_{1,2,3}\right) \end{array}\\ \phi^{\prime }\begin{array}{cc} & \left(\Delta V_{3}=\min\Delta V_{1,2,3},\phi^{\prime }>0\right) \end{array}\\ \phi^{\prime }+2\pi \begin{array}{cc} & \left(\Delta V_{3}=\min\Delta V_{1,2,3},\phi^{\prime }<0\right) \end{array}\\ \phi^{\prime }+\pi \begin{array}{cc} & \left(\text{else}\right) \end{array} \end{array}\right. \end{array}$$

From Equation (9), we can find that the estimation of orientation is independent of the size of sensor and the displacement sensing element. The root mean square error (RMSE) of estimated orientation is 1.01°.

#### 4.3.2. Estimation of Obliquity

**Figure 9.**
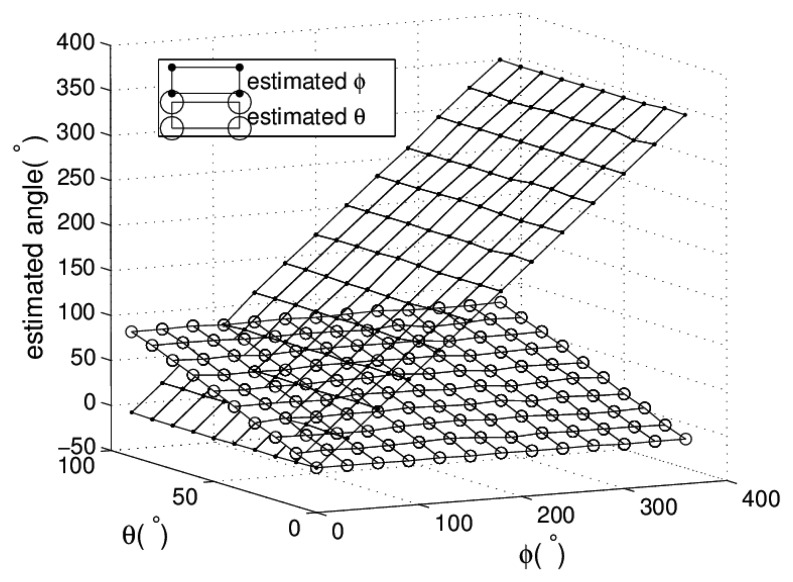
Estimated *ϕ* and *θ* on sample positions.

In Equation (6), each equation is linear to the obliquity at a fixed orientation. Its slope is the function of orientation as demonstrated in Equation (10). Results are the weighted mean of obliquities calculated by two equations of Equation (6). The RMSE of estimated obliquity is 1.22°. Figure 9 shows the estimated results of *ϕ* and *θ*.

(10) $$\begin{array}{l} k_{1}=\sqrt{3}\;\cdot \;k_{v}\;\cdot \;a\;\cdot \;\sin(\phi -\frac{\pi }{3})\\ k_{2}=\sqrt{3}\;\cdot \;k_{v}\;\cdot \;a\;\cdot \;\sin(\phi +\frac{\pi }{3})\\ \theta =(\frac{(\Delta V_{1}-\Delta V_{2})\;\cdot \;\left|k_{1}\right|}{k_{1}}+\frac{(\Delta V_{1}-\Delta V_{3})\;\cdot \;\left|k_{2}\right|}{k_{2}})*\frac{1}{\left|k_{1}\right|+\left|k_{2}\right|} \end{array}$$

#### 4.3.3. Estimation of the Additional Angular Parameter

**Figure 10.**
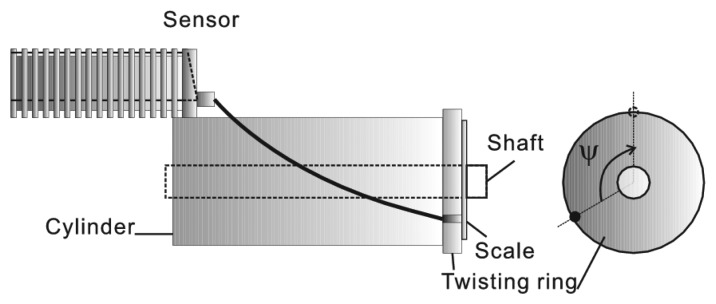
A structure imitates the measurement of torsion angle of shoulder joint.

The forearm, arm or thigh can be sketched as elliptic cylinders. Figure 10 builds a structure for imitating the measurement of the additional rotation angle of a human joint. The bundle of three wires has a spiral movement on the surface of the cylinder by the rotation of the twisting ring. The cylinder has radius in 15 mm and length in 80 mm. Δ*V*_1_, Δ*V*_2_ and Δ*V*_3_ are measured with *ϕ*, *θ* equaling to 0° and rotation angle *ψ* ranging from 0° to 180°.

**Figure 11.**
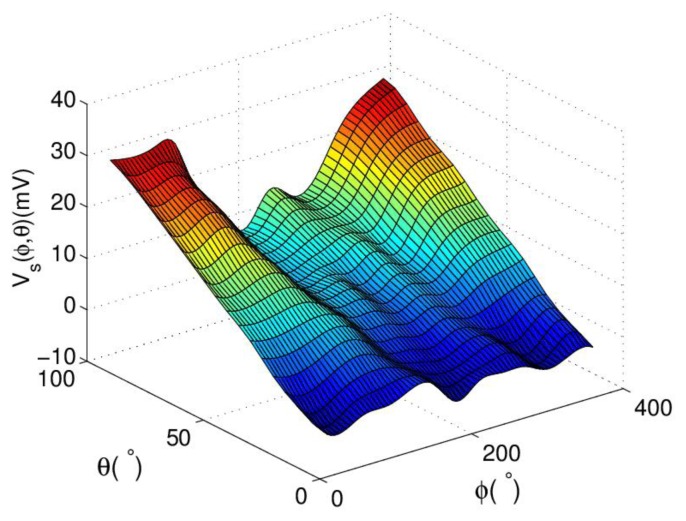
2D interpolation of *g*(*ϕ*, *θ*).

In Equation (8), the expression of *g*(*ϕ*, θ*)* is different for different individuals. Because of the multiformity of the tested objects, it is difficult to calculate the numerical expression of *g*(*ϕ*, *θ*) theoretically. 2-D cubic spline interpolation is selected as the data processing method because of its smooth peculiarity and high accuracy. The database for interpolation is calculated by the precalibration process. For the tested prototype, the interpolation samples are the experimental results achieved in basic mode. The results of 2-D interpolation on discrete grid points with 5° interval are shown in Figure 11. Substituting the estimated *ϕ*, *θ* and *g*(*ϕ*, *θ)* into Equation (8), Γ(*ψ*) can be obtained as follows:

(11) $$\begin{array}{l} \Gamma (\psi )=\frac{1}{3}(\Gamma_{1}(\psi )+\Gamma_{2}(\psi )+\Gamma_{3}(\psi ))\\ \left\{ \begin{array}{l} \Gamma_{1}(\psi )=\Delta V_{1}-g(\phi ,\theta )-k_{v}\;\cdot \;a\;\cdot \;\cos\phi \;\cdot \;\theta \\ \Gamma_{2}(\psi )=\Delta V_{2}-g(\phi ,\theta )-k_{v}\;\cdot \;a\;\cdot \;\cos(\phi -\frac{2\pi }{3})\;\cdot \;\theta \\ \Gamma_{3}(\psi )=\Delta V_{3}-g(\phi ,\theta )-k_{v}\;\cdot \;a\;\cdot \;\cos(\phi +\frac{2\pi }{3})\;\cdot \;\theta \end{array}\right. \end{array}$$

**Figure 12.**
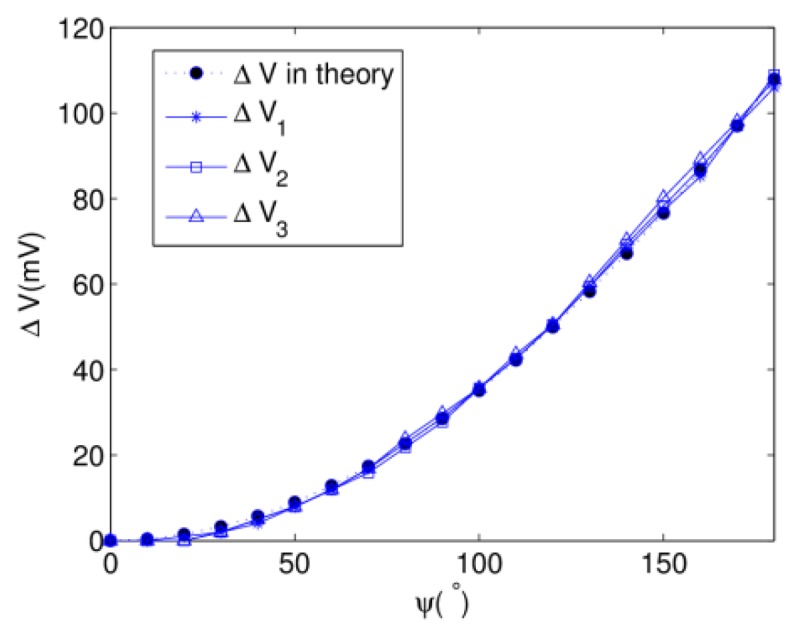
Estimation of torsion angle *ψ*.

The expression of Γ(*ψ*) can be deduced theoretically or fitted by experimental results. For the torsion experimental setup, the bundle has a spiral movement on the surface of the cylinder. Using the equation to calculate the length of spiral line, the output voltage change can be calculated theoretical as below:

(12) $$\Gamma (\psi )=\Delta V=k_{v}\;\cdot \;(\sqrt{\frac{R_{c}^{2}\psi^{2}}{d_{c}^{2}}+1}-1)$$

where *R_c_* is the radius of cylinder and *d_c_* is the length of cylinder. With rotation angle *ψ* ranging from 0° to 180°, the theoretical outputs is achieved by Equation (12) and its curve is shown in Figure 12.

For the reason that the twisting experiment is done with *ϕ* and *θ* equaling 0°, substituting into Equation (8), Δ*V*_1,2,3_ are generated only by a single variable *ψ* and are almost equal to each other. As indicated in Figure 12, the experimental curves of three potentiometers are similar to the theoretical curve and almost overlap each other. Γ(*ψ*) is the mean value of Δ*V*_1,2,3_. In a practical application, fitting the equation of Γ^−1^(*ψ*) from experimental data is more preferable because the human body is not in ideal cylinder shape.

## 5. Discussion and Conclusion

The inaccuracy of the prototype in basic mode is approximately RMSE = 1.01° in orientation, and RMSE = 1.22° in obliquity. The inaccuracy of the third angle changes with different working situations. *ϕ*, *θ* and *ψ* are reconstructed in sequence and the inaccuracy also transmits and increases in sequence. The accuracy of the proposed method is satisfactory for the reason that although the prototype is all handmade, its accuracy can reach the level of the works using high cost transducers. For example, the reported RMS of angle error in [6] is 2.5°−4.8°, RMSE = 1.3° in [8], and the flexion resolution of [6] ranges from 0.4° to 3°. Reference [22] reports the mean error of estimated flexion angles is 1.3°, and the error ranges between almost ±4°. Systematic errors are mainly due to the factors below. One is the slight distortion of the section of tube that changes the bending radius of each sensing element. The experimental setup error for setting the referenced position is 0.5°, which will also affect the accuracy of estimated results. Improving the technological manufacture level will surely increase the accuracy.

In the basic mode, the hysteresis of the tube has little effect on the estimated results because of the elimination of the integral part. Therefore, the dynamic character of the proposed device mainly depends on the sensing element used for the displacement measurement. The wire acceleration of some commercial wire-type displacement sensors reaches 10 g. The device is able to measure the high speed motion such as that of athletes by employing a high level linear displacement sensor. The direct and immediate measurement of joint angles can help calculate the velocity and the acceleration during the movement.

The measurement of orientation is full range, while the range of obliquity and the additional angle parameters can be increased freely by increasing the measurement range of the wire displacements. Use of a high level linear displacement sensor will increase the accuracy. As to practical applications, after determining the tube radius first, by considering the whole range of actual joint motion, the maximum displacement of each sensing element can be achieved by experimentation or calculation. Then we can choose the sensing elements with the corresponding range to build the device. The flexibility and big range of estimated angles is suitable for various applications.

The proposed device is simple but maybe still be somewhat cumbersome. Our future work will try to reduce its size for convenience. The soft tissues of the body may allow positioning of the sensor relative to the body to change as motion occurs. Therefore, a firm mount on human body and *in vivo* measurements are expected in future work.

The paper has proposed a multifunctional joint sensor with measurement adaptability for human gesture measurement. The sensor imitates the skin and utilizes a corrugated tube as the main body, which is flexible and contributes to the wearable character of the sensor with less restraints. Its multifunctional capability lies in its ability to simultaneously measure MDOF with a single sensor, which is designed in two working modes. The basic mode works for determining the orientation and obliquity of a 3D joint over big ranges and stands out for its applications to different joints of different individuals without recalibration. The optional advanced mode enables an additional DOF measurement to benefit a variety of applications. Although the advanced mode increases the algorithm complexity, it can reduce the physical system complexity. There are only three outputs of the proposed sensor, which is the minimum number needed for reconstructing three angular parameters. This will greatly reduce the volume of testing data compared to other approaches.

No use of inertial elements and the compact structure of the device provide the adaptability for both static and dynamic environment measurements, and both body pose and motion capture. The simple reconstruction algorithm and small output data volume are capable of providing real-time angles and long-term monitoring with environmental universality. The performance assessment of the built prototype is promising enough to indicate the feasibility of the sensor.
